# Early Peritoneal Immune Response during *Echinococcus granulosus* Establishment Displays a Biphasic Behavior

**DOI:** 10.1371/journal.pntd.0001293

**Published:** 2011-08-30

**Authors:** Gustavo Mourglia-Ettlin, Juan Martín Marqués, José Alejandro Chabalgoity, Sylvia Dematteis

**Affiliations:** 1 Cátedra de Inmunología, Departamento de Biociencias, Facultad de Química, Universidad de la Republica, Montevideo, Uruguay; 2 Laboratory of Vaccine Research, Departamento de Desarrollo Biotecnológico, Instituto de Higiene, Facultad de Medicina, Universidad de la Republica, Montevideo, Uruguay; George Washington University, United States of America

## Abstract

**Background:**

Cystic echinococcosis is a worldwide distributed helminth zoonosis caused by the larval stage of *Echinococcus granulosus*. Human secondary cystic echinococcosis is caused by dissemination of protoscoleces after accidental rupture of fertile cysts and is due to protoscoleces ability to develop into new metacestodes. In the experimental model of secondary cystic echinococcosis mice react against protoscoleces producing inefficient immune responses, allowing parasites to develop into cysts. Although the chronic phase of infection has been analyzed in depth, early immune responses at the site of infection establishment, e.g., peritoneal cavity, have not been well studied. Because during early stages of infection parasites are thought to be more susceptible to immune attack, this work focused on the study of cellular and molecular events triggered early in the peritoneal cavity of infected mice.

**Principal Findings:**

Data obtained showed disparate behaviors among subpopulations within the peritoneal lymphoid compartment. Regarding B cells, there is an active molecular process of plasma cell differentiation accompanied by significant local production of specific IgM and IgG2b antibodies. In addition, peritoneal NK cells showed a rapid increase with a significant percentage of activated cells. Peritoneal T cells showed a substantial increase, with predominance in CD4^+^ T lymphocytes. There was also a local increase in Treg cells. Finally, cytokine response showed local biphasic kinetics: an early predominant induction of Th1-type cytokines (IFN-γ, IL-2 and IL-15), followed by a shift toward a Th2-type profile (IL-4, IL-5, IL-6, IL-10 and IL-13).

**Conclusions:**

Results reported here open new ways to investigate the involvement of immune effectors players in *E. granulosus* establishment, and also in the sequential promotion of Th1- toward Th2-type responses in experimental secondary cystic echinococcosis. These data would be relevant for designing rational therapies based on stimulation of effective responses and blockade of evasion mechanisms.

## Author Summary

Cystic echinococcosis is a zoonotic disease caused by the larval stage of the cestode *Echinococcus granulosus* and shows a cosmopolitan distribution with a worldwide prevalence of roughly 6 million infected people. Human cystic echinococcosis can develop in two types of infection. Primary infection occurs by ingestion of oncospheres, while secondary infection is caused by dissemination of protoscoleces after accidental rupture of fertile cysts. Murine experimental secondary infection in Balb/c mice is the current model to study *E. granulosus*-host interaction. Secondary infection can be divided into two stages: an early stage in which protoscoleces develop into hydatid cysts (infection establishment) and a later stage in which already differentiated cysts grow and eventually become fertile cysts (chronic infection). During infection establishment parasites are more susceptible to immune attack, thus our study focused on the immunological phenomena triggered early in the peritoneal cavity of experimentally infected mice. Our results suggest that early and local Th2-type responses are permissive for infection establishment.

## Introduction

Helminths are metazoan parasites currently infecting a quarter of the world population [1]. The high prevalence of helminthiasis reflects one outstanding feature of parasite infections: chronicity. This fact could be read into helminths having special skills to adapt to defense mechanisms triggered by infected hosts, in order to survive for long periods of time. Thus it is not surprising that in most cases host immune responses are ineffective in parasite elimination. Chronicity observed in the context of parasite infections often associates with polarized cytokine responses. In this respect, although helminths belong to a highly divergent animal group, they induce polarized and stereotyped Th2-type responses, with rare to no levels of Th1-type components [2].

Cystic echinococcosis is a zoonotic disease caused by the larval stage of the cestode *Echinococcus granulosus*, and shows a cosmopolitan distribution with a worldwide prevalence of roughly 6 million infected people [3]–[5]. Parasite cysts are able to live for very long periods of time within the infected host, and thus it is thought that *E. granulosus* evades or modulates the host immune response through still unknown mechanisms. In this regard, the experimental model of secondary infection has been used to study host-parasite interactions. This model is based on the intraperitoneal (ip) inoculation of viable protoscoleces into susceptible and immunocompetent mice [6]. Using Balb/c mice strain, secondary cystic echinococcosis can be divided into two stages: an early stage (until day 20–30 pi) in which the infection establishes (protoscoleces develop into hydatid cysts) [7], followed by a late or chronic stage in which already differentiated cysts grow and eventually become fertile cysts. There is scarce information regarding early immune responses in the peritoneal cavity of infected mice [8]–[10]. Breijo *et al*. by means of ip implanted diffusion chambers found that the initially triggered local inflammation resolves and completely disappears by day 30 pi [11]. These results suggest that infection establishment is associated with a strong local control of inflammation during the initial phase of protoscoleces differentiation into hydatid cysts.

Cytokines key roles during *E. granulosus* experimental infection were first analyzed by Rogan [12]. Focusing on chronic infections, the author suggested that systemic Th2-type cytokine responses would be an actively induced mechanism used by the parasite in order to suppress the expression of potentially harmful Th1-type cytokines [12]. Regarding early stages of infection, Dematteis *et al*. showed that there is an early and systemic Th2-type cytokine response which is unrelated to protective immunity [13]. On the other hand, immune mechanisms associated with IFN-γ effects seem to be relevant to the development of protective immune responses [14], [15]. Therefore, it has been suggested that the development of an early Th2-type cytokine response could be read into a mechanism modulated and/or actively induced by the parasite to favor its survival. Due to the lack of information about early and local immune responses in experimentally infected mice, the present work reports a deep and detailed analysis of early cellular and molecular immunological phenomena taking place in the peritoneal cavity of infected mice.

## Materials and Methods

### Ethics statement

Animal experiments were performed in compliance with Comisión Honoraria de Experimentación Animal (CHEA) - Universidad de la República according to the Canadian Guidelines on Animals Care and the National Uruguayan Legislation No.18.611 since 2009 (“Animales en Experimentación e Investigación”). Protocols were approved by Comité de Ética - Facultad de Química - Universidad de la República (Uruguay) and were given the Protocol Approval Number 030510 (http://csic1.csic.edu.uy/chea).

### Mice, parasites and experimental infections

Adult female Balb/c mice were obtained from DILAVE (Uruguay) and housed at the animal facility of Instituto de Higiene (Montevideo, Uruguay). Protoscoleces were obtained by aseptic puncture of fertile bovine hydatid cysts, and were washed several times with phosphate buffered saline (PBS) pH 7.2 containing gentamicin (40 µg/mL) [13]. Protoscoleces viability was determined by means of eosin exclusion and flame cell activity [13]. Only those batches with over 90% viability were used. For experimental infections, mice were inoculated ip with 2000 viable protoscoleces in 200 µL of PBS (infected mice), or 200 µL of PBS alone (control mice).

### Peritoneal cells isolation

At different times post-inoculation (pi), infected and control mice (numbers are shown under each figure) were sacrificed by cervical dislocation prior inhalatory anesthesia. Peritoneal cavities were extensively washed with cold RPMI 1640 supplemented with 2% FCS. Red blood cells were lysed by treatment with RBC Lysing Buffer (Sigma) and remaining leukocytes were counted using Neubauer chamber and trypan blue staining. Individual control mice were always highly homogeneous, thus they were grouped to increase sample space for statistical analyses.

### Flow cytometry

Cell suspensions were pre-incubated with anti-mouse CD32/CD16 mAb (Mouse Fc Block, Becton Dickinson) for 30 min at 4°C and then were incubated during 30 min at 4°C with the following anti-mouse MoAb: CD19-PE, CD19-FITC, CD3-PE, CD3-FITC, CD4-FITC, CD8-FITC, panNK-PE, CD138-PE, CD25-PE y CD69-FITC, and their specific isotype controls (Pharmingen, Becton Dickinson). Cellular viability was analyzed by propidium iodide staining (Pharmingen, Becton Dickinson). Acquisitions and analyses were performed using a FACScalibur flow cytometer (Becton Dickinson) and Cell Quest® software (Becton Dickinson), respectively.

### qRT-PCR

RNA extraction was performed using TRIzol® (Invitrogen) and DNA contamination was eliminated by DNasa I treatment (Invitrogen) following manufacturer's recommendations. cDNA was then obtained by 1 µg RNA reverse transcription using M-MLV-RT (Invitrogen) at 42°C for 50 min. qPCR reactions were performed using mouse specific primers available under request for FoxP3, Pax5, Blimp-1, Bcl-6, TNFα, IFN-γ, IL-2, IL-12_p35, IL-15, IL-4, IL-5, IL-6, IL-10, IL-13, TGF-β and β-actin. qPCR were performed using QuantiTect SYBR Green PCR Kit (QIAGEN) following manufacturer's instructions and 0.9 µM of each specific primer in a Rotor-Gene 6000 (Corbett Life Science). The cycling used for every reaction was 95°C for 15 min, 40 cycles at 95°C for 15 sec and 60°C for 1 min, followed by a melting curve rising from 72°C to 90°C. β-actin was used as a normalizing gene. Relative mRNA amounts were calculated using the 2^−ΔΔCt^ method [16], and samples fold increase/decrease was referred to control group values.

### Specific antibodies

Peritoneal cells from 5-days infected mice were cultured without stimulus in complete RPMI 1640 (10% heat inactivated FCS, 100 µg/mL streptomycin, 100 U/mL penicillin, 10 mM L-glutamine and 50 µM 2-mercaptoethanol) during 72 h at 37°C and 5% CO_2_. Anti-PSA (protoescolex somatic antigens) specific antibodies were measured by ELISA in individual supernatants according to Dematteis *et al*. [17]. In equally diluted samples, specific IgM, IgG1, IgG2a, IgG2b and IgG3 were determined using appropriate goat or rabbit anti-(mouse Ab isotype) antibodies labeled with peroxidase (Sigma). Peroxidase activity was detected using *O*-phenylendiamine as chromophore (Sigma), and absorbance values were recorded at 492 nm.

### Statistics

Statistical analyses were assessed by non-parametric Mann-Whitney U test and differences were regarded as significant with *p*<0.05.

## Results

### Peritoneal lymphocytes show disparate behaviors at very early stages of experimental infection

**Figure 1:**
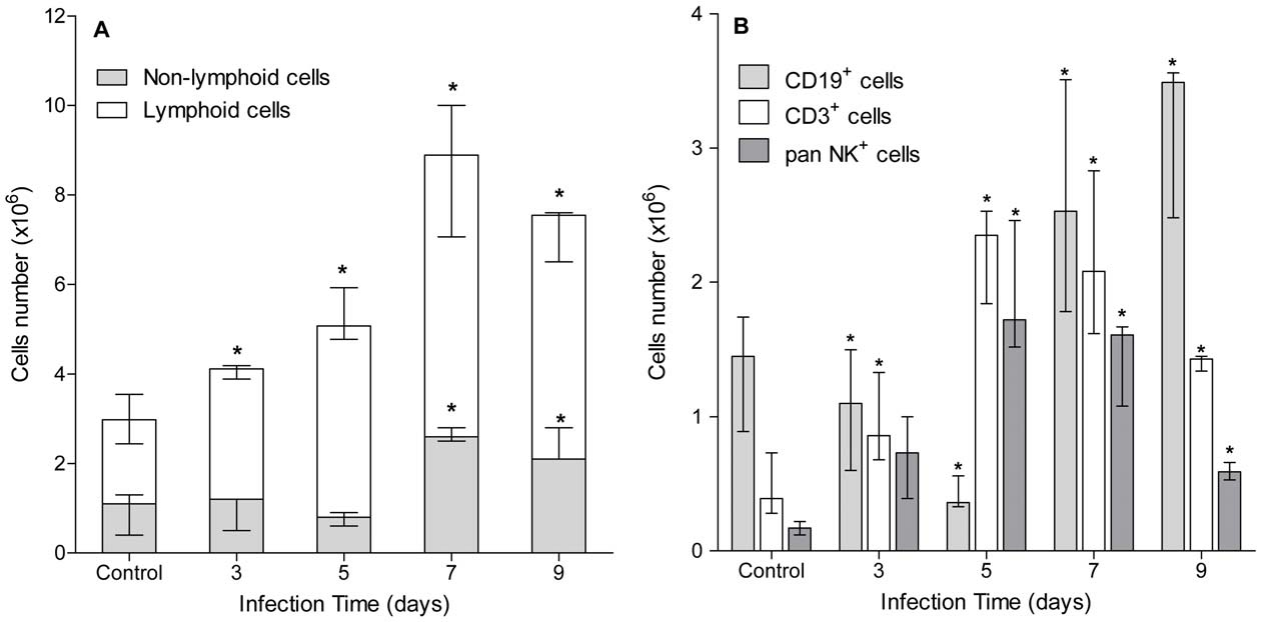
Peritoneal lymphocytes show disparate behaviors at very early stages of experimental infection. A group of mice (n = 12) was inoculated ip with 2000 protoscoleces and another group (n = 8) was inoculated with equal volume of sterile PBS (control group). Three infected and 2 control animals were sacrificed at days 3, 5, 7 and 9 pi and their total peritoneal leukocytes, lymphocytes and non-lymphoid cells (A), and T (CD3+), B (CD19+) and NK (panNK+) lymphocytes (B) were analyzed by flow cytometry. Results are shown as group median and data range. (*) Statistical significance (p<0.05) compared to control group. Results are representative of two independent experiments.

Firstly, we analyzed peritoneal cells behavior in Balb/c infected mice. It is worth mentioning that some parasite-adhered peritoneal cells (e.g. macrophages) may have been unintentionally discarded due to technical limitations of peritoneal washings. Thus, whenever peritoneal cells are mentioned throughout this work, we strictly refer to parasite-non-adhered peritoneal cells. Results in Figure 1.A. show that peritoneal leukocytes number rapidly increased, reaching a 3-fold peak by day 7 pi. Then, by means of size (FSC) and complexity (SSC) flow cytometry parameters we analyzed the contribution of lymphocytes and non-lymphoid cells to this behavior. Non-lymphoid cells number remained unchanged until 5 days pi and showed a 2-fold increase by day 7 pi (Figure 1.A.). Contrarily, peritoneal lymphocytes number showed an early and significant increase by day 3, which was sustained until day 9 pi. Because of such peritoneal lymphocytes behavior, we further analyzed the kinetics of the three main lymphocyte populations. Figure 1.B. shows disparate behaviors among peritoneal lymphocytes. Both T cells (CD3^+^) and NK cells (pan NK^+^) number rapidly increased by day 3 pi, peaking at day 5–7 pi (Figure 1.B.). On the contrary, B cells (CD19^+^) behavior was very different. CD19^+^ cells number significantly decreased by day 5 pi, and then recovered reaching a 2-fold increase by day 7–9 pi (Figure 1.B.). Overall, these results show that infection by *E. granulosus* induces significant and early changes mainly in the peritoneal lymphoid compartment of infected mice.

### Peritoneal B cells drop is associated with a plasma cell differentiation process

**Figure 2:**
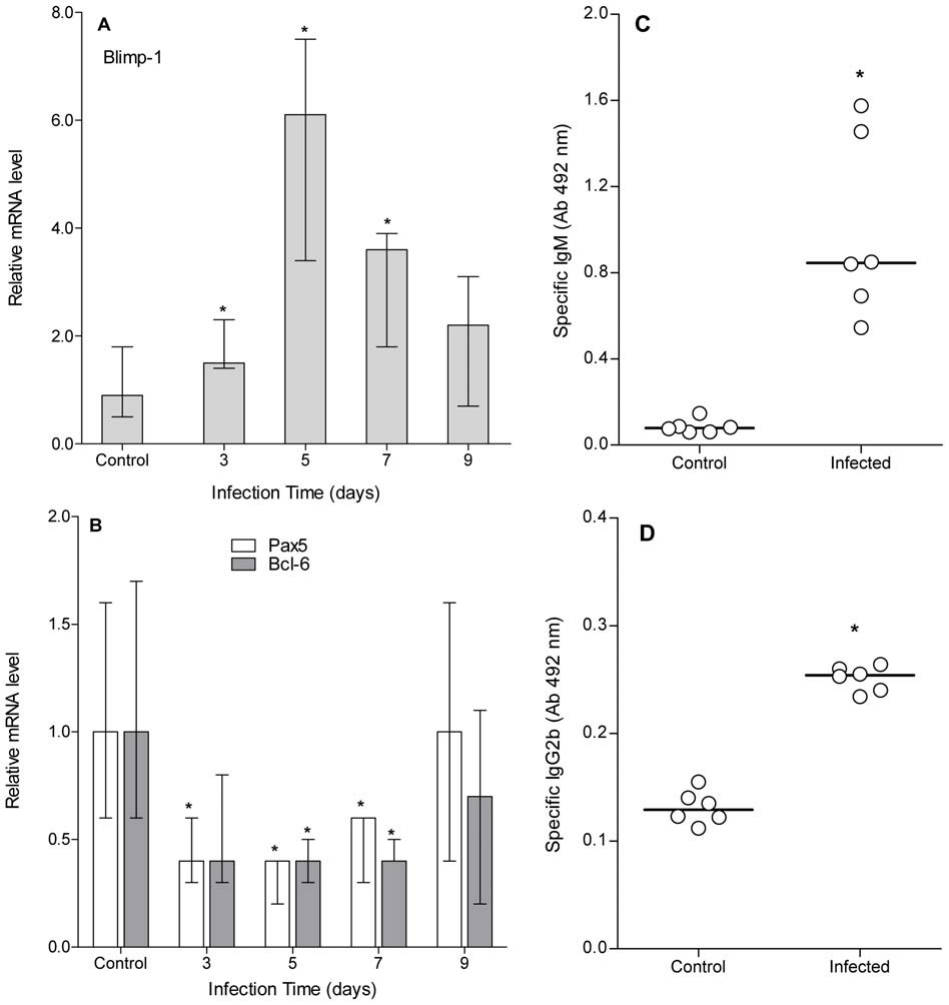
Peritoneal B cells drop is associated with a plasma cell differentiation process. A group of mice (n = 12) was inoculated ip with 2000 protoscoleces and another group (n = 8) was inoculated with equal volume of sterile PBS (control group). Three infected and 2 control animals were sacrificed at days 3, 5, 7 and 9 pi and their peritoneal cells were recovered. qRT-PCR was performed using specific primers for murine Pax5, Blimp-1 and Bcl-6, and relative mRNA levels were expressed respect to control group. Results are shown as group median and data range (A and B). Another group of infected (n = 6) and control mice (n = 6) were sacrificed at day 5 pi and their peritoneal leukocytes were culture for 72 h in complete RPMI without stimulation. Anti-PSA specific IgM, IgG1, IgG2a, IgG2b and IgG3 titers were determined by ELISA in culture supernatants. Results are shown as individual values (circles) and group median (lines) (C and D). (*) Statistical significance (p<0.05) compared to control group. Results are representative of two independent experiments.

Initial analyses, aimed at explaining possible causes of B cells decrease, dismissed massive cell death phenomena (no propidium iodide staining among peritoneal cells) and terminally differentiated plasma cells (CD19^−^CD138^+^ cells) (data not shown). Thus, we further analyzed in depth the presence of local antibody secreting cells (ASC). Qualitative flow cytometry analyses showed a quick rise in large and CD19^low^ lymphocytes (data not shown), suggesting the existence of a local ASC differentiation process [18], [19]. Because ASC differentiation is tightly regulated at the molecular level by specific transcription factors, we next analyzed Pax5, Bcl-6 and Blimp-1 local expression at different time points. Results in Figure 2.A. and 2.B. show a consistent expression profile related to ASC differentiation. Additionally, functional evidence of local ASC was obtained by analyzing specific antibodies titers in culture supernatants of non-stimulated peritoneal cells from 5-days infected mice. Interestingly, only IgM and IgG2b anti-PSA titers were increased (Figure 2.C. and 2.D.). Overall, results reported here reveal the existence of a peritoneal ASC differentiation process in early stages of infection, which is characterized by large CD19^low^ cells and an active transcriptional program of plasma cell differentiation. Moreover, local ASC are a source of specific IgM and IgG2b antibodies.

### Peritoneal NK cells show a significant rise with a proportion of activated cells

**Figure 3:**
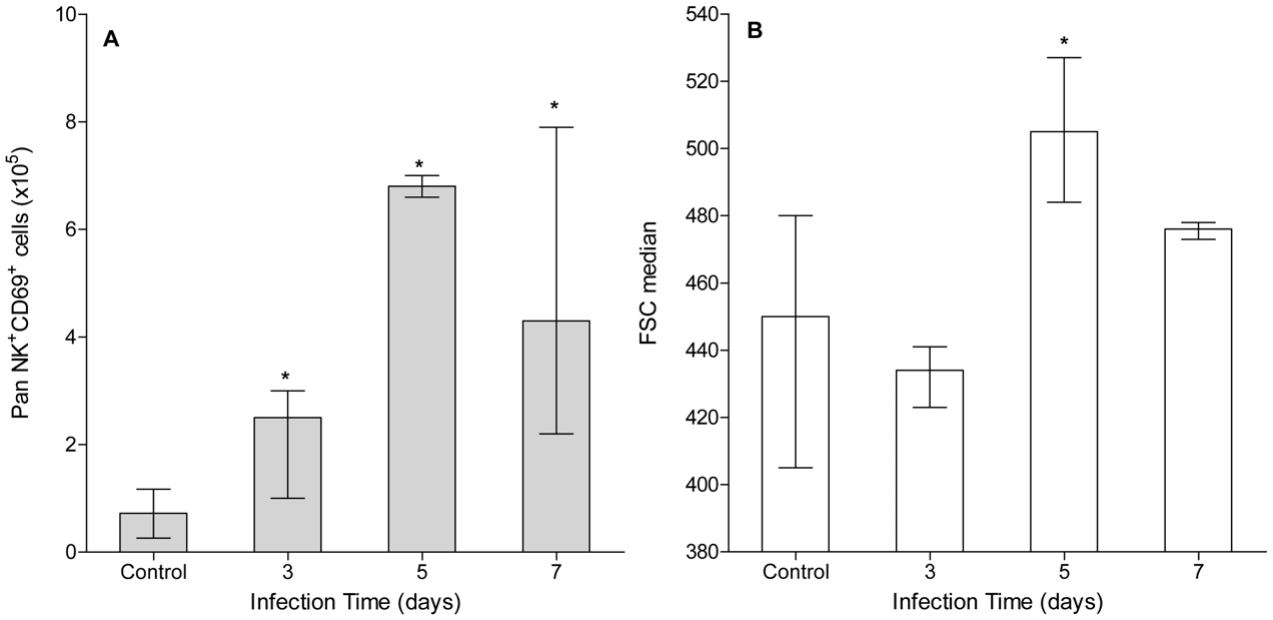
Peritoneal NK cells show a significant rise with a proportion of activated cells. A group of mice (n = 9) was inoculated ip with 2000 protoscoleces and another group (n = 6) was inoculated with equal volume of sterile PBS (control group). Three infected and 2 control animals were sacrificed at days 3, 5 and 7 pi and their peritoneal activated NK cells (A) as well as median cellular size (B) were determined. Results are shown as group median and data range. (*) Statistical significance (p<0.05) compared to control group. Results are representative of two independent experiments.

Information regarding NK cells role in helminth infections is scarce and partially controversial. In the experimental model of *E. granulosus* infection there has been no reports to our knowledge on NK cells behavior so far. Phenotypic characterization of peritoneal NK cells showed a rapid increase in activated NK cells (CD69^+^panNK^+^ cells) peaking at day 5 pi (Figure 3.A.). Although this value represents a 12-fold increase respect to control animals, it is interesting to note that only a 40% of peritoneal NK cells showed an activated phenotype. Since activated NK cells increase their cellular size [20], we also analyzed it by flow cytometry observing a significant increase in median FSC values on peritoneal NK cells at day 5 pi (Figure 3.B.).

### Peritoneal CD4^+^, CD8^+^ and Treg cells augment in early stages of infection

**Figure 4:**
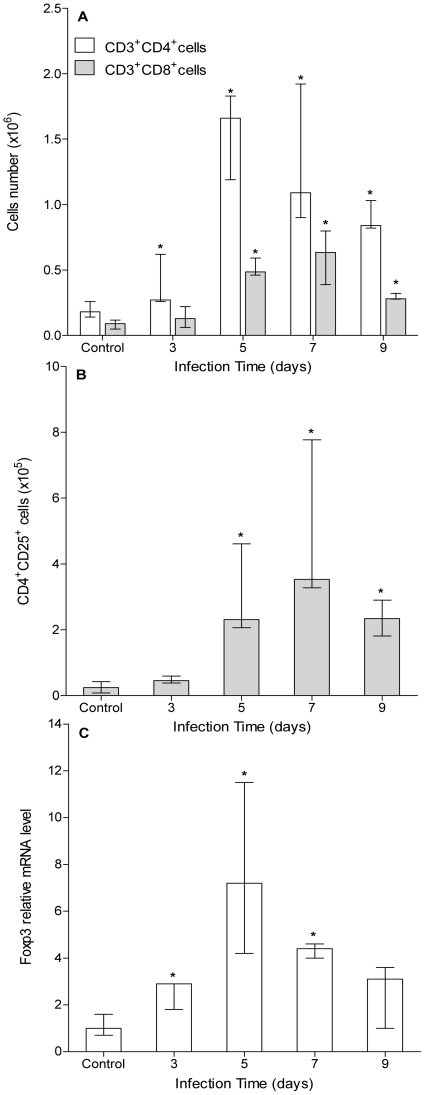
Peritoneal CD4^+^, CD8^+^ and Treg cells augment in early stages of infection. A group of mice (n = 12) was inoculated ip with 2000 protoscoleces and another group (n = 8) was inoculated with equal volume of sterile PBS (control group). Three infected and 2 control animals were sacrificed at days 3, 5, 7 and 9 pi and their peritoneal CD4+ and CD8+ T cell subsets composition (A) and CD4+CD25+ T cells (B) were analyzed by flow cytometry. Results on phenotypic analyses are representative of two independent experiments. Foxp3 expression was performed in peritoneal cells from infected and control mice using specific primers, and relative mRNA levels were expressed respect to control group (C). Results are shown as group median and data range. (*) Statistical significance (p<0.05) compared to control group.

Peritoneal T lymphocytes (CD4^+^ and CD8^+^) were also analyzed. Kinetic analyses reported here showed a rapid increase in CD3^+^CD4^+^ cells by day 3 pi reaching an 8-fold increase by day 5–7 pi, and a slower increase in CD3^+^CD8^+^ cells from day 5 pi reaching a 6-fold peak by day 7 pi (Figure 4.A.). We also analyzed the presence of Treg cells within the CD4^+^ T cells peritoneal compartment. Results shown in Figure 4.B. indicate a 15-fold increase in CD4^+^CD25^+^ T cells by days 5–7 pi. It is well known that CD4^+^CD25^+^ phenotype is not exclusive of Treg cells, being also shared by activated CD4^+^ T cells [21], [22]. Therefore, to confirm the local and early presence of Treg cells we further analyzed Foxp3 mRNA expression level. Figure 4.C. shows a rapid and significant increase in Foxp3 transcripts from day 3 pi, peaking by day 5 pi. To our knowledge, this is the first report on Treg cells within the peritoneal cavity of *E. granulosus* infected mice. Overall, results reported here suggest an active role for peritoneal T cells subpopulations during early experimental infection by *E. granulosus*.

### Peritoneal cytokine response shows an early biphasic behavior

**Figure 5:**
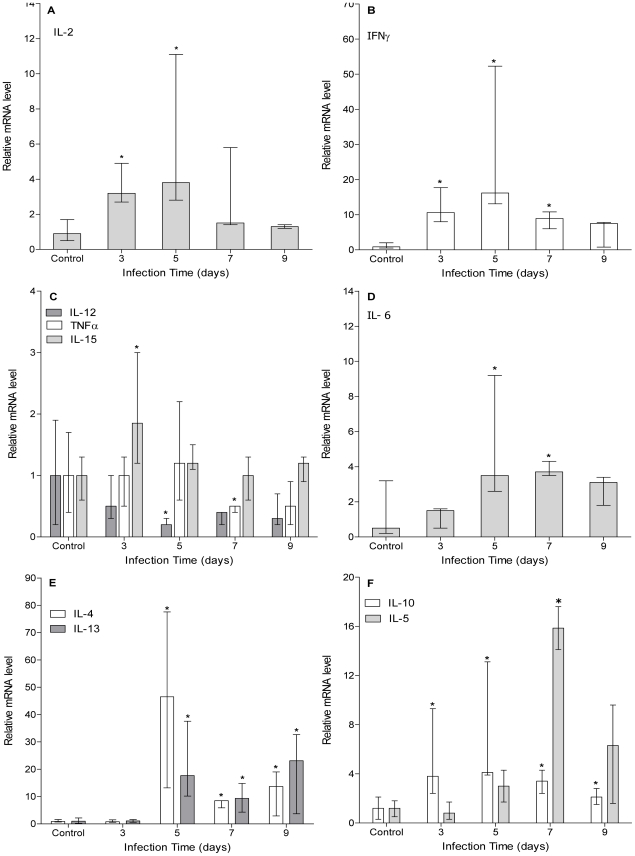
Peritoneal cytokine response shows an early biphasic behavior. A group of mice (n = 12) was inoculated ip with 2000 protoscoleces and another group (n = 8) was inoculated with equal volume of sterile PBS (control group). Three infected and 2 control animals were sacrificed at days 3, 5, 7 and 9 pi and their peritoneal cells were recovered. qRT-PCR was performed using specific primers for murine IL-2 (A), IFN-γ (B), IL-15, IL-12 and TNFα (C), IL-6 (D), IL-4 and IL-13 (E), IL-5 and IL-10 (F). Relative mRNA levels are expressed respect to control group. Results are shown as group median and data range. (*) Statistical significance (p<0.05) compared to control group.

Although cytokine responses during murine *E. granulosus* infection have been analyzed to some extent, early and local cytokine profile in infected mice has not been described. Hence, we performed a qRT-PCR analysis for several cytokines in peritoneal cells from mice at early stages of infection. Expression analysis of Th1-type associated cytokines (TNFα, IFN-γ, IL-2, IL-12 and IL-15) showed that IFN-γ, IL-2 and IL-15 are quickly induced (Figure 5.A., 5.B. and 5.C.). Their expression levels were significantly higher than control animals as soon as 3 days pi. IL-15 transcript levels returned to baseline values by day 5 pi, whereas IFN-γ and IL-2 induction was sustained until day 5 of infection. In contrast, IL-12 and TNFα showed a slight but significant decrease in their transcript levels at day 5 and 7 pi, respectively (Figure 5.C.). On the other hand, Th2-type cytokines (IL-4, IL-5, IL-6, IL-10 and IL-13) were induced later in time (Figure 5.D., 5.E. and 5.F.). Expression levels of IL-4, IL-6 and IL-13 increased significantly from day 5 pi, while expression of IL-5 did not augment until day 7 pi. Interestingly, IL-10 showed a sustained induction over time from day 3 pi until the end of the experiment. TGF-β did not increase its transcript levels throughout the analyzed time points (data not shown). Results reported here show a biphasic kinetic behavior in cytokine expression profile. Th1-type cytokines seem to predominate from infection beginning until day 5 pi followed by a switch towards a Th2-type response. These results are in accordance with previously obtained data using pooled peritoneal cells from infected and control mice at each time point (our own unpublished results).

## Discussion

Studies regarding early stages in natural secondary infections are virtually absent from current literature. Although ip inoculation of protoscoleces into immunocompetent mice is a dissimilar situation from natural secondary infections, due to differences mainly in the immune status of hosts with a primary infection, this model may pose at least some hypotheses on the early interaction between protoscoleces and the immune system.

Early and local immune responses elicited during experimental secondary infection by *E. granulosus* have been scarcely studied [8]–[11]. Susceptibility of *E. granulosus* protoscoleces to immune effectors is thought to be greater during early stages of infection, being only a small percentage of parasites able to develop into defined hydatid cysts [14], [23]–[25]. Protoscoleces become cysts in a relative short period of time [7], thus we focused our work on the characterization of early immunological events taking place at the anatomical site of infection establishment, e.g. peritoneal cavity.

Peritoneal leukocytes number in infected mice increased quickly due to proliferation of resident cells and/or systemic recruitment (Figure 1.A.). Non-lymphoid cells number showed no early changes. However, it is worth noting that analyses were performed from day 3 pi and non-lymphoid cells are usually recruited very rapidly, tending to disappear just as fast. For example, in experimental infections by *Brugia malayi* and *B. pahangi* neutrophils are the main peritoneal population 24 h pi, completely disappearing by day 3 [26], [27]. Therefore, results reported here may not completely exclude earlier (e.g. neutrophils) or later (e.g. macrophages) roles for non-lymphoid cells during *E. granulosus* establishment in the murine model of secondary cystic echinococcosis.

In contrast, peritoneal lymphocytes showed a very different behavior (Figure 1.B.). While B cells number dropped at day 5 pi, T and NK cells number increased from day 3 peaking both populations at days 5–7 pi. These results are very different to those reported for experimental *Taenia crassiceps* infection, where peritoneal lymphocytes peak at 6–8 weeks of infection [28]. Therefore, this conflicting lymphocyte kinetics in similar models of ip cestode infections suggests that lymphocytes behavior might be largely dependent on parasite species.

Peritoneal B cells were shown to undergo an early plasma cell differentiation process in *E. granulosus* infected mice (Figure 2). Plasma cell differentiation induces migration to anatomical sites of final maturation and survival niches, probably explaining the local drop in B cells counts [19]. Plasma cell differentiation is a tightly regulated molecular process; characterize by up- and down-regulation of specific transcription factors [29]. Pax5 and Bcl-6 keep mature B cells in its naïve state by suppressing (among other functions) the expression of Blimp-1, the master transcription factor for plasma cell differentiation. Such process can be evidenced by decreased levels of Pax5 and Bcl-6, with a concomitant increase in Blimp-1 expression. Our analyses proved the existence of an active molecular process of plasma cell differentiation during the early stages of experimental infection, which was functionally confirmed by the local secretion of anti-PSA IgM and IgG2b antibodies (Figure 2). Such isotype profile was similar to that observed in T-independent humoral responses [30]. In fact, similar results have been described in *E. granulosus* infected CD4KO mice at the systemic level [31]. Interestingly, CD40^−/−^ mice infected with *E. multilocularis* produce specific IgM, IgG2b and IgG3 antibodies after 2 months of infection [32]. Thus, early T-independent antibody responses could be a common phenomenon in experimental infection by *Echinococcus spp*. Moreover, regarding the isotype restriction reported here it is interesting to note that tegument and suckers of *E. granulosus* protoscoleces have Fc-like receptors able to bind human IgG1 and IgG3 antibodies trough their specific Fc portions [33]. Such isotypes structurally and functionally correspond to murine IgG2a and IgG2b, respectively [34]; and therefore local IgG2b specific induction in early stages of infection could be seen as a possible evasion strategy exploited by *E. granulosus*.

NK cells have been shown to play major roles in early infections by viruses, bacteria and protozoa [35]–[37]. However, there are very few reports referring to their role in helminth infections [38]–[42]. Here, we have shown that peritoneal NK cells not only rapidly rise in number but also display an activated phenotype (Figure 3). Interestingly, the peak in NK cells number (day 5 pi) is associated with a 40% of cells exhibiting an activated phenotype. Our results are in agreement with reports on murine *Litomosoides sigmodontis* natural infection showing local expansion and activation of NK cells [41]. Although *E. granulosus* and *L. sigmodontis* infections are dissimilar systems at the parasitological and immunological levels, such a common behavior in NK cells suggests that they may play important roles in helminth infections.

Peritoneal T cells analyses showed differential kinetics among T cells subsets (Figure 4). CD4^+^ T cells number showed a faster increase than CD8^+^ T cells, being the former the main contributor to total T cells increase. Rises in the number of local CD4^+^ T cells have been described in several helminth infections [43], [44]. However, there are very few reports regarding CD8^+^ T cells behavior. For example, *E. multilocularis* chronically infected humans display an increase in oligoclonality and activation status of their CD8^+^ T cells [45]. Similarly, murine chronic infections by *E. granulosus* have shown rises in splenic CD8^+^ T cells [46], and *E. multilocularis* protoscoleces are able to induce suppressor CD8^+^ cells in naïve splenocytes cultures [47]. Some reports have also shown that locally expanded CD8^+^ T cells seem not only to play inefficient roles in helminth infections, but also are probably responsible for suppressive immune responses [48],[49]. On the other hand, CD4^+^CD25^+^Foxp3^+^ Treg cells have been shown to play key roles in animals and humans with helminth infections [50]–[54]. Experimental data have verified an old hypothesis stating that helminths actively suppress or reduce immunopathological conditions in their hosts. These effects are now partially attributed to recruitment and/or induction of CD4^+^CD25^+^Foxp3^+^ Treg cells [53]. Results reported here are an initial and rough characterization of Treg cells behavior in the peritoneal cavity of early *E. granulosus*-infected mice, showing a nice correlation between Foxp3 expression and CD4^+^CD25^+^ T cells (Figure 4). Although this is the first report on peritoneal Treg cells in early infection by *E. granulosus*, Mejri *et al*. have recently reported the presence of a subpopulation of peritoneal CD4^+^CD25^+^ Treg cells in *E. multilocularis* infected mice [55]. It is worth noting that while they reported an increase in peritoneal Treg in 6-weeks infected mice, our results have shown a much earlier rise (5–7 days pi). Overall, these results suggest that CD4^+^CD25^+^Foxp3^+^ Treg cells could be involved in the modulation of immune responses within the peritoneal cavity of *Echinococcus spp.* infected mice.

Initial studies on the early immune response in *E. granulosus* infected mice showed a defined Th2-type systemic cytokine profile, suggesting that immune polarization is an early event [13]. Here, we have shown that the expression of some Th1-type cytokines is quickly induced at the local level (Figure 5). However, the decline observed for IL-12 and TNFα transcripts suggests that the initial inflammatory response is weak. In addition, the absence of IL-12 induction could explain the short duration of the Th1-type cytokine profile because IL-12 is a key mediator in early Th1-type bias [56]. Interestingly, this set of cytokines seems to be related to peritoneal NK cells behavior because they are factors involved in their proliferation (IL-15), activation (IL-2) and effector functions (IFN-γ) [57]. Early NK cells increase and activation phenotype could be partially explained by the quick IL-15 and IL-2 local induction. Moreover, the highest number of activated NK cells correlated well with the greatest increase in IL-2 and IFN-γ transcripts. Regarding IL-2, it also has been described as an important autocrine factor for T cells proliferation (including Treg cells), being therefore a probable explanation for the early peritoneal T cells rise [56]. On the other hand, Th2-type cytokines showed a later induction (Figure 5) except for TGF-β which did not increase its transcript levels (data not shown). TGF-β activity has been shown not to be dominantly regulated by classical transcriptional (expression) and/or translational (production) means, but mainly through enzymatic conversion of latent TGF-β expressed on cell surfaces [58]. Thus, TGF-β analyses through qRT-PCR not always derive in conclusive results. IL-4, IL-6 and IL-13 transcripts significantly increased from day 5 pi, and IL-5 did it only at day 7 pi. Interestingly, IL-10 showed a sustained induction from day 3 of infection. Most of these cytokines (IL-4, IL-5, IL-6 and IL-10) are involved in plasma cell differentiation [19], and their induction correlated well with B cells drop and plasma cell transcription factors profile. Moreover, early IL-10 induction could be attributed to the rapid increase in local Treg cells, because they are well known to secrete large amounts of IL-10 [59]. Regarding Treg cells, it is worth noting that Foxp3 expression increased at day 5 pi (Figure 3.C.) as well as IL-6 (Figure 5.D.). Although IL-6 has been shown to impair Foxp3 induction in Treg cells [60], it has been also reported that this is not always the case at least *in vitro* [61]. On the other hand, our results showed that Foxp3 induction precedes IL-6 up-regulation. Indeed, Foxp3 transcripts are already up-regulated at day 3 pi, peaked at day 7 and then down-regulated its mRNA levels (Figure 4.C.), while IL-6 is induced at day 5 pi being its transcripts level quite constant thereafter (Figure 5.D.). Quickly up-regulated IL-10 (Figure 5.F.) could be responsible for the early Foxp3 induction, which shuts itself down once IL-6 is up-regulated. Thus, our results are not absolutely conclusive about the relationship among cytokine expression profiles and Treg cells behavior.

Results reported here have clearly shown biphasic kinetics in local cytokines expression. While Th1-type cytokines initially prevail and return to baseline values by day 7 pi, Th2-type cytokines are later induced (day 5 pi) and become predominant by day 7 pi. Our results are in accordance with those reported for murine cysticercosis due to *T. crassiceps* infection [62]. In early stages of experimental cysticercosis a clear but transient Th1-type immune response develops (high levels of IL-2 and IFN-γ), but as infection time progresses a permanent Th2-type response follows (high levels of IL-4, IL-6 and IL-10). Thus, authors suggested that the sequential activation of Th1- and Th2-type responses would favor parasite reproduction [62]. In secondary cystic echinococcosis, the early switch from a Th1- to a Th2-type local cytokine response, could favor developing protoscoleces to reach the immune resistant cystic stage.

**Figure 6:**
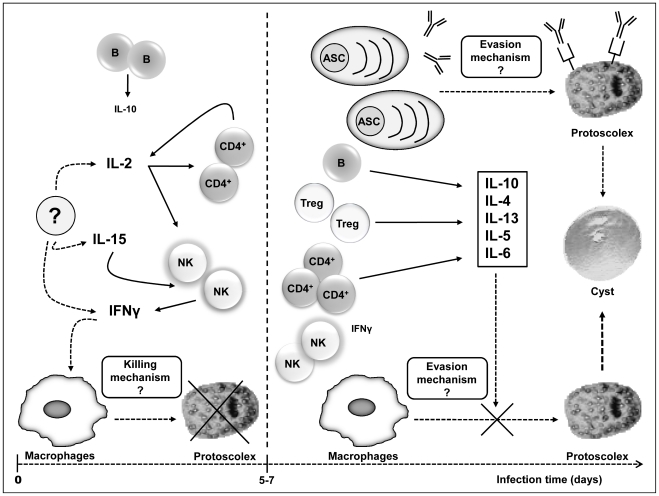
Early immunological phenomena in the peritoneal cavity of infected mice. After ip inoculation of protoscoleces into Balb/c mice approximately only 10% of parasites finally develop into hydatid cysts. Therefore, early immune responses although not optimal to prevent infection establishment, are probably not entirely ineffective. Based on our results, we suggest that NK cells may play an important role in very early infection. Activated NK cells through IFN-γ production could be partially responsible for the activation of resident macrophages inducing their protoscolicidal activity. IFNγ-activated macrophages as well as local antibodies recognizing protoscolex antigens and able to activate the complement system could be partially responsible for the elimination of 90% of parasite inoculum. From day 5 pi *E. granulosus* protoscoleces would bias the cytokine response towards a Th2-type profile and induce/recruit cells with regulatory activity (Treg). These phenomena would block initially triggered effects by IFN-γ. Moreover, antibody isotype restriction (IgG2b and IgM) could be seen as a possible evasion strategy exploited by *E. granulosus* since protoscoleces have Fc-like receptors able to bind specific isotypes trough their Fc portions. Overall, this schematic representation shows a possible explanation for the reasons of infection establishment in mice.

Finally, our results pose a possible explanation for the early and local events in *E. granulosus* experimental infection (Figure 6). It has been well documented that approximately only 10% of inoculated protoscoleces finally develop into defined hydatid cysts [14], [23]–[25]. Therefore, early immune responses although not optimal to prevent infection establishment, are probably not entirely ineffective. Based on preliminary results obtained from infected Balb/c mice treated with polyI:C we hypothesized that NK cells could play an important role in very early infection (our own unpublished results). Activated NK cells through IFN-γ production could be partially responsible for the activation of resident macrophages inducing their known protoscolicidal activity [14]. We are currently performing several experiments in order to verify such hypothesis. On the other hand, it has been reported that in the presence of specific antibodies on the protoscoleces surface, the tegumental membrane depolarization due to complement activation is faster and stronger than that observed in the absence of specific antibodies [63]. Therefore, IFNγ-activated macrophages as well as local antibodies recognizing protoscolex antigens and able to activate the complement system could be partially responsible for the elimination of approximately 90% of parasite inoculum. From day 5–7 pi *E. granulosus* would be able to bias the cytokine response towards a Th2-type profile and induce/recruit cells with regulatory activity (e.g. Treg cells). These phenomena would block the initial effects triggered by IFN-γ, allowing at last the infection establishment. It is worth highlighting the absence of early IL-12 induction since it has been shown that forced IL-12 production in experimentally infected Balb/c mice negatively influence the infection outcome, suggesting that Th1-type responses could generate some protective effects [15].

In conclusion, our results open new ways to investigate the involvement of several immune effector players in *E. granulosus* protoscoleces killing, and also in the sequential promotion of Th1- towards Th2-type immune responses in the model of secondary cystic echinococcosis.
